# Supplementary material for: TmSpz-like Plays a Fundamental Role in Response to E. coli but Not S. aureus or C. albican Infection in Tenebrio molitor via Regulation of Antimicrobial Peptide Production
Source: Int J Mol Sci. 2021 Oct 8;22(19):10888. doi: 10.3390/ijms221910888 (PMC8509142; doi:10.3390/ijms221910888)
Supplement: Supplementary file 1 [file ijms-22-10888-s001.zip › ijms-1368513-supplementary.pdf]

International Journal of
Molecular Sciences

MDPI

Supplementary Materials

*Tm*Spz-like Plays a Fundamental Role in Response to *E. coli* but Not *S. aureus* or *C. albican* Infection in *Tenebrio molitor* via Regulation of Antimicrobial Peptide Production

Ho Am Jang [1], Bharat Bhusan Patnaik [2], Maryam Ali Mohammadie Kojour [1], Bo Bae Kim [1], Young Min Bae [1], Ki Beom Park [1], Yong Seok Lee [3], Yong Hun Jo [1,*] and Yeon Soo Han [1,*]

1 Department of Applied Biology, Institute of Environmentally-Friendly Agriculture (IEFA), College of Agriculture and Life Sciences, Chonnam National University, Gwangju 61186, Korea; hoamjang@gmail.com (H.A.J.); maryam.alimohammadie@gmail.com (M.A.M.K.); kbb941013@gmail.com (B.B.K.); ugisaka@naver.com (Y.M.B.); misson112@naver.com (K.B.P.)

2 P. G. Department of Bio-Science and Biotechnology, Fakir Mohan University, Balasore 756089, OD, India; drbharatbhusan4@gmail.com

3 Department of Biology, College of Natural Sciences, Soonchunhyang University, Asan City 31538, Korea; yslee@sch.ac.kr

* Correspondence: yhun1228@jnu.ac.kr (Y.H.J.); hanys@jnu.ac.kr (Y.S.H.)

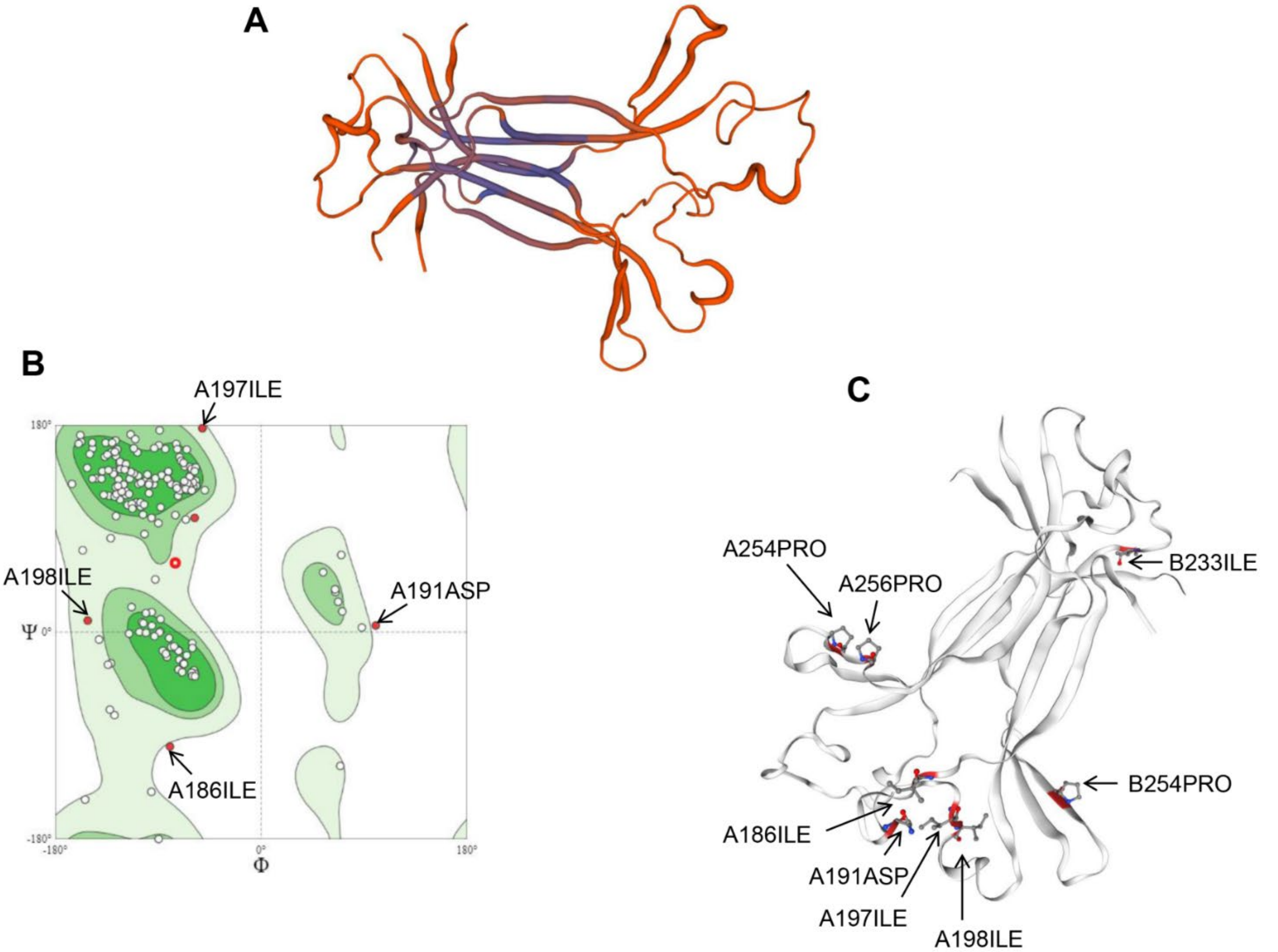

Figure S1. Predicted model of *Tm*Spz-like using the SWISS-MODEL program. (A) Homology model of *Tm*Spz-like based on the reference model [SMTL ID: 3e07.1]. Crystal structure of Spätzle cysteine knot homodimer. (B) Ramachandran plot diagram of *Tm*Spz-like that allows a combination of conformational angles psi (ψ) and phi (φ). The shaded region

corresponds to allowed conformation of residues. Ramachandran outlier residues are shown in the predicted model. (C) Ramachandran outliers (A-chain: 198ILE, 197ILE, 191ASN, 250PRO, 256PRO; B-chain; 233ILE, 254PRO) constitute 3.94% of the residues of *Tm*Spz-like.

Table S1. Percentage amino acid sequence identity of *Tm*Spz-like with its orthologs in other insects

	TcSpz	PxSpz	BiSpz	NlSpz	ClSpz	HhSpz	ArSpz-like	DmSpzXJ	DmSpzXI	AaSpz1B	PvSpz5-like	SeSpz	MsSpz1A	MsSpz1B
TmSpz-like	85	38	49	43	43	43	40	36	36	36	35	34	26	26
TcSpz	100	36	49	42	40	42	39	35	35	33	35	37	28	28
PxSpz		100	40	41	38	42	45	30	30	34	31	41	43	43
BiSpz			100	48	47	40	47	28	28	32	37	36	31	31
NlSpz				100	51	48	67	35	35	35	33	37	33	33
ClSpz					100	52	47	37	37	41	37	40	41	41
HhSpz						100	43	33	33	38	39	33	36	36
ArSpz-like							100	28	28	35	33	40	34	34
DmSpzXJ								100	100	34	25	30	32	32
DmSpzXI									100	34	25	30	32	32
AaSpz1B										100	30	27	30	30
PvSpz5-like											100	36	29	29
SeSpz												100	52	52
MsSpz1A													100	100
